# *In Vitro* Cell Models for Ophthalmic Drug Development Applications

**DOI:** 10.1089/biores.2016.0008

**Published:** 2016-04-01

**Authors:** Sara Shafaie, Victoria Hutter, Michael T. Cook, Marc B. Brown, David Y.S. Chau

**Affiliations:** ^1^ Department of Pharmacy, Pharmacology, and Postgraduate Medicine, The Research Center in Topical Drug Delivery and Toxicology, School of Life and Medical Sciences, University of Hertfordshire, Hertfordshire, United Kingdom.; ^2^ MedPharm Ltd., Guildford, Surrey, United Kingdom.

**Keywords:** cell culture, drug delivery, ophthalmology, tissue engineering, toxicity testing

## Abstract

Tissue engineering is a rapidly expanding field that aims to establish feasible techniques to fabricate biologically equivalent replacements for diseased and damaged tissues/organs. Emerging from this prospect is the development of *in vitro* representations of organs for drug toxicity assessment. Due to the ever-increasing interest in ocular drug delivery as a route for administration as well as the rise of new ophthalmic therapeutics, there is a demand for physiologically accurate *in vitro* models of the eye to assess drug delivery and safety of new ocular medicines. This review summarizes current existing ocular models and highlights the important factors and limitations that need to be considered during their use.

## Introduction

**FIG. 1.**
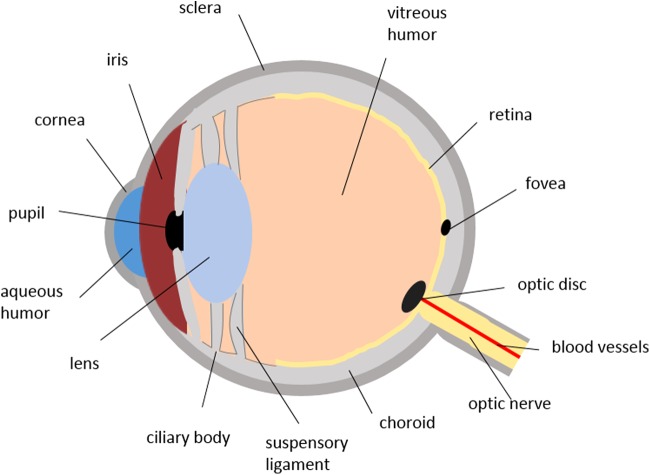
Schematic diagram of the human eye.

The human eye, a fluid-filled sphere, is a unique and highly protected organ with a complex anatomy and physiology, containing several specialized structures with distinct physiological functions. It is mainly divided into two parts: the anterior and the posterior segments. The anterior segment comprises the cornea, conjunctiva, iris, ciliary body, aqueous humor, and lens, and the posterior segment contains the sclera, choroid, retina, and vitreous humor^1^ (Fig. 1).

In the anterior and posterior segments of the eye, ocular barriers play an important part in controlling and regulating the inward and outward transport of solutes, fluids, and also administered drugs. Upon topical administration to the anterior part of the eye, drug molecules are prevented from reaching their ocular site of action as a result of anterior static barriers (i.e., tight junctions of corneal epithelium [CE] and blood–aqueous barriers) and also anterior dynamic barriers (i.e., lacrimal drainage and tear fluid barrier, conjunctival blood and lymphatic clearance). In addition, posterior ocular tissue hinders drug permeation due to expression of efflux pumps on the cell membrane and also by the presence of static (i.e., sclera, retinal pigment epithelium [RPE], and blood capillary endothelial cells) and dynamic barriers (i.e., choroidal blood and lymph circulation).^2^ Therefore, the administered drug is often required to overcome such ocular barriers before reaching its site of action. For many years, *in vivo* animal models have been used in ophthalmic studies, mainly to evaluate the level of irritation and toxicity of administered molecules to the ocular cells and tissue. However, in recent years, due to cost, time, and ethical issues associated with animal use,^3,4^ researchers have been encouraged to develop alternative techniques for ocular investigations. These techniques include *ex vivo* models of deceased animal tissue and *in vitro* human and animal cell culture models. The aim of this review is to provide an overview of the established and recently constructed ocular models and the advantages and limitations associated with these models.

## Ocular Models

### *In vivo/ex vivo* models

Animal experimentation plays an important role in the research and development of ophthalmic drugs and ocular delivery systems.^3^ For many years, live animals have been utilized to assess the effect of various ocular products to the eye.^5,6^ The rabbit is known as the most commonly used animal model with larger animals such as pigs, monkeys, dogs, and cats being less frequently used. In addition, the value of mice and rats are limited in ocular studies due to their small eye size.^3^

Following permanent eye injuries caused by a cosmetic dye sold in the 1930s,^7^ the FDA developed the rabbit *in vivo* Draize test for evaluating acute ocular toxicity.^8^ Draize test is an international standard assay in which New Zealand white rabbits are mostly used as they are readily obtainable, relatively inexpensive, and have a well-described anatomy with large eyes.^6^ In this protocol, 0.1 mL of the test substance is applied onto only one eye of the conscious rabbit, whereas the untreated eye serves as a control.^8^ After 72 h exposure of the test substance on the cornea, conjunctiva, and iris, chemicals can be classified on a subjective scoring, which ranges from nonirritating to severely irritating.^5^

Despite its gold standard status and being the only validated test for evaluating irritation severity in full range, the Draize test has been criticized for numerous limitations, including its time consuming and subjective nature of assessment, its lack of repeatability and reproducibility,^9^ high dosage of test materials used,^10^ variable estimation of results, and overprediction of human response,^11^ which is mainly related to interspecies differences.

Consequently, the Draize test has been modified both in protocol and data analysis from its original form.^12^ In 1980, Griffith and colleagues developed the low volume eye irritation test (LVET), as an alternative animal method and following a recommendation from the National Research Council.^13^ In 1977, the National Research Council committee suggested that the Draize test drawbacks might be more of a volume–response correlation rather than a species–response difference between rabbits and humans. LVET is an alteration to Draize testing in which test substances are only applied to the corneal surface of the rabbit's eye and at a lower volume (0.01 mL vs. 0.1 mL). The rationale in reducing the instilled volume is that it is more representative of the lacrimal fluid volume of both the human and the rabbit eye. Therefore, the LVET was described to cause less stress to tested rabbits and also results could better predict human ocular irritation response.^11^ However, results obtained following exposure to severe irritants in LVET were considered to be an underestimation of results in comparison with the Draize data.^14^ Therefore, it is debatable whether to accept LVET as a more accurate test as it lacks the element of exaggeration and overprediction of human responses present in Draize testing.^15–18^ This, on the other hand, raises concerns over assuring public safety due to its moderate protocol.^12,19^ As a result, it is still criticized for using animals and it has yet to be accepted as an alternative test by regulatory agencies.

More recently, ocular organotypic models (Table 1) have been used to minimize the use of live animals in experimental studies. These isolated ocular systems retain physiological and biochemical functions of the mammalian enucleated eye or cornea.^20,21^ Opacity and permeability of the isolated cornea under the effect of a test substance is quantitatively measured using opacitometry and spectrophotometry, respectively. These measurements combined with histological analysis evaluate the extent of damage caused by the test substance, and subsequently drive an eye irritation classification for prediction of potential *in vivo* ocular irritation of a test substance.^5,22^

**Table 1.** ***Ex Vivo* Organotypic Models Used in Ocular Testing**

Name	Test method indicator	Testing objective	Validation status	Limitations	References
Bovine corneal opacity and permeability (BCOP)	Increase in corneal thickness, permeability, and opacity	Ocular sensitivity and corrosion	EVCAM statement of scientific validity for identification of severe irritants and ocular corrosives	Not as sensitive in distinguishing between mild irritants with the standard protocol	^22,34^
Isolated chicken eye (ICE)	Increase in corneal thickness, permeability, and opacity	Ocular sensitivity and corrosion	EVCAM statement of scientific validity for identification of severe irritants and ocular corrosives	Possible limitation for solids	^22,34^
Isolated rabbit eye (IRE)	Increase in corneal thickness, and opacity	Ocular sensitivity and corrosion	Further review is recommended	Possible limitation for solids	^22,34^

EVCAM, European Center for the Validation of Alternative Methods.

Burton et al. developed the first ocular organotypic model known as the isolated rabbit eye (IRE) test method.^23^ The IRE, also known as rabbit enucleated eye test, was originally used to detect irreversible eye damage caused by severe irritants.^24^ IRE protocols have developed over time and have been widely assessed by regulatory bodies (e.g., The European Commission/British Home Office).^5^ In 1997, an evaluation of the test concluded that the assay lacks the ability to predict irritation over the full range and can only be useful for evaluating severe irritants.^25^ To date, IRE is mainly used for nonregulatory optimization studies as it is not characterized as a valid assay for ocular irritancy classification.^26^ In response to the deficiencies associated with IRE, Prinsen and Koëter developed the isolated chicken eye (ICE) test method.^27^ Chicken eyes are readily available from slaughter houses with consistent quality and dimensions that make them a practical replacement for IRE. Toxic responses are measured by changes in opacity, fluorescein retention, thickness of tissue upon swelling, and assessment of changes related to the surface of the tissue.^28^

In addition, in 1992, Gautheron et al. developed the bovine cornea opacity and permeability (BCOP) assay based on methods originally developed by Muir^29^ and Tchao.^30,31^ The BCOP assay was internationally accepted in 2009 and its scientific suitability is recognized in identifying substances that can cause serious damage as well as substances categorized as nonirritants.^5^ Using porcine cornea in BCOP is advantageous as it more accurately resembles human cornea in terms of thickness and structure and has also been frequently used in ocular studies.^32^ These models have been able to generate promising results with fewer ethical concerns and at reduced costs. However, they all share the mutual drawback that anatomical and physiological differences among interspecies are still associated with these tests. In addition, these models are only limited to evaluating the corneal effects of the substances and not the systemic effects. In 2007, the scientific advisory committee of the European Center for the Validation of Alternative Methods (ECVAM) issued a statement on organotypic *ex vivo* assays as ocular screening tests to detect possible corrosives and severe irritants. Based on this statement, both BCOP and ICE test methods are scientifically valid to identify severe ocular irritants, whereas validation of the IRE method required additional work to be performed and further review was recommended.^33^

Tissue viability is prolonged when the culture medium is periodically refreshed to retain sufficient levels of supplements and to eliminate metabolic waste products from the cellular environment. Recently, bioreactors that perfuse medium have enabled organotypic models to retain cell viability over an extended period of time.^35^ Bioreactors are commonly described as devices in which development of biological and/or biochemical processes are under closely controlled environmental and operating conditions (e.g., temperature, pH, oxygen, pressure, medium flow rate, pressure, nutrient supply, and waste metabolite elimination).^36,37^ Perfusion culture systems in bioreactors have shown to improve and prolong cell viability survival while maintaining cellular functionality.^38^ Thuret et al. used an innovative bioreactor for storage of *ex vivo* cornea, which maintained intraocular pressure and continuously renewed medium. This allowed rapid reduction of stromal swelling and improvement of endothelial cell viability in comparison to the corneal immersion in a sealed flask.^39^

## *In Vitro* Cell-Based Models

Limitations associated with *in vivo* models have encouraged development and validation of various alternative *in vitro* models derived from animal and human primary and immortalized cells. The exploitation of appropriate *in vitro* models is crucial for the development of new approaches to overcome ocular barriers. In comparison with *in vivo* and *ex vivo* models, *in vitro* cell-based models offer the advantage of being simple, quick to construct, relatively inexpensive, and reproducible, while providing mechanistic understanding of the results.^3,10^ In addition, *in vitro* models can be used to evaluate a number of combinations of experimental parameters, which is often not achievable with animal models.^40,41^ These models are commonly used for basic science, pharmaceutical research and development, toxicology, and permeability studies. The use of both primary and immortalized cell culture models of ocular barriers is described in the literature. However, *in vitro* models based on primary cells have limited cell division and growth in culture media, do not retain cell characteristics beyond three or four passages, and may also differ from isolate to isolate. Therefore, the emphasis on various areas of ocular investigation has been on the development of *in vitro* models based on animal and human immortalized cell lines.^12^

Primary cells can be transformed using chemicals or viruses to establish continuous/immortalized cells. With immortalized cell lines, there is no longer a need for the long process of tissue isolation, which is followed by primary cell harvesting and cell purification. Furthermore, cells in culture are stable over a greater passage number with the ability to rapidly expand in growth medium if a large number of cells are required for experiments. However, immortalized cells may have altered growth characteristics, become tumorigenic, and secrete abnormal levels of proteases and cell surface markers. Moreover, expression of many differentiated or tissue-specific enzymes can be decreased and it is more likely for them to express chromosomal abnormalities.^42^

### Corneal morphology

**FIG. 2.**
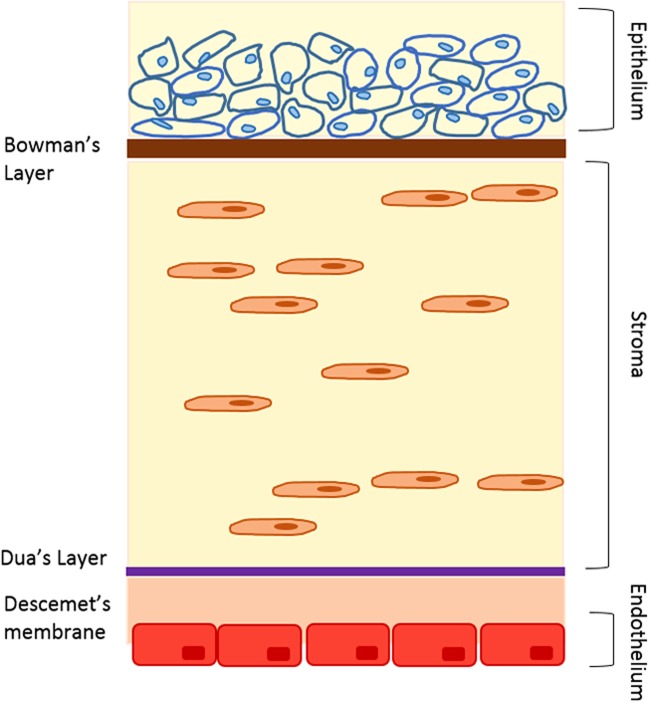
Schematic presentation of different layers of the cornea.

The cornea is the outermost part of the anterior segment of the eye and is always in direct contact with the external environment.^43^ The transparent and avascular human cornea contains six layers (Fig. 2), and has an average horizontal diameter of about 11.5 mm, a vertical diameter of 10.5 mm, and an average radius of curvature of 7.7 mm curvature that remains constant throughout life.^44,45^ About 90% of the corneal thickness is made up of 200–250 uniformly spaced collagenous lamellae that intersect and run parallel to the surface to cover the complete extent of the cornea. This layer is called stroma, which offers corneal transparency and physical strength.^46^ Five more layers make up the remaining 10% of the cornea, which are the epithelium and Bowman's layer at the front of the cornea and Dua's layer, Descemet's membrane, and the endothelium at the back of the cornea.^47^

The cornea is the major route of absorption for topically applied drugs, and CE tight junctions are the most apical intercellular junctions that play an important role in the establishment and maintenance of corneal barrier function.^48^ The uppermost layers of CE cause over 60% of corneal transepithelial electrical resistance (TEER) and CE is known as the rate-limiting barrier for transcorneal permeation. Therefore, models of the cornea are very useful in studying drug permeation, absorption, and ocular toxicity. To date, cell cultured corneal models range from simple monolayers to stratified epithelium, to cocultures of epithelium and stroma, and to more complex three-dimensional (3D) tissue-engineered corneal equivalents.^49^

### Corneal models

#### Corneal epithelial models

The CE is the outermost layer of the eye, which acts as a major barrier of the eye. Since the 1960s, many *in vitro* epithelial models have been established as replacements to Draize test based on primary and immortalized cell lines of human and animal origin.^50^

#### Primary models

Most of the primary cell culture models (Table 2) that mimic corneal barrier are constructed from isolated corneal epithelial cells from rabbits.^3^ For instance, Kawazu et al. established a primary rabbit corneal model which resembled the morphology of an intact cornea and was used for studying permeability of beta adrenergic antagonist and levofloxacin^51–53^; however, the model's TEER and tight barrier were not comparable to that of cornea *in vivo.*^54,55^ In another study by Chang et al., a primary rabbit corneal epithelial membrane was developed with more distinct barrier properties due to air-interface culture conditions.^56^ Models of human primary epithelial cells have also been investigated as toxicology models.^5,57^ Furthermore, human primary corneal epithelial (HCE) cell has been used for preparation of tissue sheets to reconstruct the ocular surface in severe ocular surface disorders.^58,59^ Some authors, such as Ban et al., were successful in developing a stratified epithelium by culturing human corneal limbal cells on human amniotic membrane, which showed tight barrier junctions after 28 days in culture.^60^ However, the application of HCEs has been limited as alternative *in vitro* models of corneal barriers due to the low availability of donor corneas.

**Table 2.** **Summary of the Corneal Epithelial Models**

Species	Application(s)	References
Primary		
Rabbit	Active transport studies and permeability	^51–53^
Rabbit	Permeability studies	^56^
Human	Ocular irritation, toxicity, and drug absorption	^79–81^
Immortalized		
SIRC	Corneal drug metabolism and transport	^65^
EpiOcular^™^	Ocular sensitivity and corrosion	^73^
SkinEthic^™^	Ocular sensitivity and corrosion	^74^
Clonetics	Ocular irritation and transepithelial permeability studies	^72^

SIRC, Statens Seruminstitut rabbit corneal cells.

### Immortalized models

#### Animal

Various models of immortalized animal corneal epithelial cells (Table 2) have been developed from the rabbit,^61,62^ rat,^63^ and hamster.^64^ Models of immortalized rabbit CE cells are more commonly developed and used compared with other animals. In addition, a rabbit corneal cell line known as Statens Seruminstitut rabbit corneal cells is widely used, despite showing a fibroblast phenotype, which in turn decreases its value as a valid model for CE.^65^ Although these models have helped in reducing the use of animals, the issues related with their nonhuman origin are still present.

#### Human

To overcome this limitation, since early 1990s, considerable efforts have been focused on developing *in vitro* models derived from human cells.^66–69^ Recently, a large amount of research is being done in the field of 3D tissue engineering models.^70,71^ Examples of the *in vitro* human-derived CE models that are commercially available as a 3D human cornea equivalents include: EpiOcular^™^ (MatTek), HCE (SkinEthic), and Clonetics (Lonza). EpiOcular is a stratified, squamous epithelial model in which a permeable polycarbonate membrane is used to culture human epidermal keratinocytes from neonatal human foreskin.^72,73^ Having an air–liquid interface gives these models the advantage of exhibiting closer morphological and growth characteristics to that of *in vivo* conditions.^22^ Similar to the EpiOcular model, HCE model of SkinEthic^™^ is developed from immortalized human CE mucosa cells cultured at the air–liquid interface using a polycarbonate substrate membrane. This model is structurally very comparable to the corneal mucosa of human eye due to the lack of stratum corneum in the air–epithelial tissue.^74^ Both EpiOcular and SkinEthic are currently used as an eye irritation test in place of the Draize test for product development.^69^ Finally, Clonetics, a model of human CE cells cultured on permeable membrane supports and provides useful information on assessing corneal penetration of ophthalmic drugs and transepithelial permeability studies.^75^ In this model, the lifespan of primary human corneal cells is extended by infecting the cells with a recombinant retrovirus.^76^

In terms of model validation, both EpiOcular and SkinEthic HCE models have undergone prospective validation by European Union Reference Laboratory for alternatives to animal testing (EURL-ECVAM) and Cosmetics Europe to distinguish potential irritants from nonirritants.^77,78^ The EpiOcular model was found to be acceptable only for the liquids and the protocol for testing the solids required further optimization. It is now accepted for differentiating irritants from nonirritants, however, EpiOcular model is not intended to distinguish between categories of irritation. In addition, to date, the SkinEthic HCE model is also validated for the testing of liquids protocol, however solids protocol is still undertaking additional optimization.^78^ Since these models are developed based on noncorneal and immortalized cell lines, there are some differences between them and intact native human cornea, which should be taken into account. For instance, primary mechanical or enzymatic detachment from corneal tissue would result in an induced traumatic stimuli and a various range of responses from the cell.^9^ Consequently, this may affect the integrity of cellular structure and cell differentiation. In addition, the immortalization process followed by culturing conditions may also alter gene expression; hence the use of immortalized cell lines may not always truly correlate with *in vivo* behavior of corneal cells.^5^ Additionally, due to the fragile nature of epithelial models, they should be handled with great care to prevent cells from drying or being damaged. *In vitro* cell-based assays also lack the presence of hormonal, immune, and neural influences, as well as cell–cell interactions. Although this will result in a less complex model, it can also be a disadvantage for an organ as specialized and complex as the eye since the interactions occurring throughout the whole tissue are not taken into account.^22^

#### Corneal equivalents

**FIG. 3.**
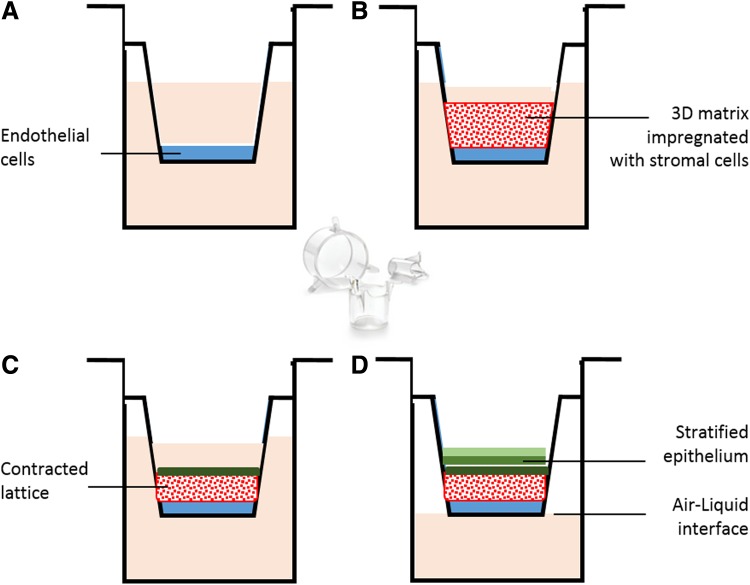
Schematic presentation of a corneal equivalent *in vitro* culture model: **(A)** Corneal endothelial cells are grown to confluency on a culture insert. **(B)** A collagen layer containing stromal cells is grown on top of the corneal endothelial cell layer. **(C)** Epithelial cells are seeded on top of the collagen layer. **(D)** Exposure to air–liquid interface results in a stratified epithelium.^71^

Due to the limitations associated with *in vitro* corneal epithelial models, several groups have attempted to develop more complex multicellular corneal equivalents. In comparison with models consisting of only corneal epithelial cells, multicellular models better replicate cellular interplay and characteristics of native cornea.^5^ The first model of a human corneal equivalent from immortalized human corneal cells was developed by Griffith et al.^82^ This model is composed of a thin layer of endothelial cells cultured on a tissue culture dish with keratocytes and support proteins in the middle covered with a layer of epithelial cells. The stromal matrix was further improved from the original study for understanding recovery mechanisms^83^ and nerve–target cell interactions.^84^ Moreover, Reichl et al. (2004) developed a bioengineered human cornea construct for permeation studies containing immortalized endothelium and immortalized stratified epithelium on top derived from HENC and CEPI 17 Cl 4, respectively with native stromal cells (fibroblasts) in a collagen hydrogel matrix (Fig. 3). Three different model drugs were used to test the barrier properties of this cornea equivalent model, such as pilocarpine hydrochloride, befunolol hydrochloride, and hydrocortisone.^71^ In addition to human corneal equivalents, there are a few models of complete cornea that have been derived from animal cells. The first *in vitro* model of the whole cornea was developed by Minami et al. derived from isolated bovine endothelial, stromal, and endothelial cells in a collagen gel matrix.^85^ Cornea-specific keratin was expressed by epithelial cells and the epithelium was a 5–6 layer of superficial cells with microvilli, wing, and basal cells. In addition, Zieske et al. have demonstrated feasibility of developing an *in vitro* model of all three corneal layers from primary rabbit CE, keratocytes, and a mouse endothelial cell line.^86^ Consequently, *in vitro* models of the epithelium, fibroblasts, and endothelium from pig and bovine were constructed for cytotoxicity testing of chemicals and surfactants.^87,88^ Unfortunately, all multicellular *in vitro* models do not exhibit the complexity of the native organ^89^ and factors such as the composition of the aqueous humor, tear fluid, and tear flow^90^ are not taken into account.

## Conjunctival Morphology

The conjunctival epithelium is a transparent mucous membrane containing only two to three cell layers and lines the inner eyelid and anterior sclera to the edge of the cornea. It serves as a protective barrier for the ocular surface, generates mucus glycoproteins (mucins) to facilitate eye lubrication and aids adherence of tear films. The conjunctiva is permeable to drugs of different size and polarity^91,92^ and plays an important part in ocular and systemic absorption of topically administered ophthalmic medications through the noncorneal route.^3,93^ For a drug to cross the conjunctiva, a significant portion of it is lost to the systemic circulation through the noncorneal route. This intraocular route of drug entry is more applicable to large and hydrophilic molecules that are poorly absorbed through the cornea. The remaining part of the drug can diffuse through sclera, which mainly contains collagen, and unlike conjunctiva, is poorly vascularized. *In vitro* models of conjunctival epithelium (Table 3) are useful tools in understanding approaches to modulate ocular noncorneal and systemic drug absorption.^3^

**Table 3.** ***In Vitro* Conjunctival Models Derived from Primary Cells**

Species	TEER (Ω cm^2^)	Application(s)	References
Primary			
Rabbit	∼1900	Active transport studies and permeability	^94–99^
Rabbit	∼1100	Transport studies and metabolism	^100–102^
Cow	∼5600	Toxicity studies	^103^
Immortalized			
Conjunctival (HCjE) cell line		Ocular surface defence mechanism	^107^

TEER, transepithelial electrical resistance.

## Conjunctival Models

To date, conjunctival epithelial cells have been mostly derived from rabbit primary cells as submerged culture models^94–99^ and the newer air–liquid interface cell culture systems.^100–102^ Recently, cell culture models of conjunctiva were developed from primary bovine conjunctival cells^103^ and immortalized rat cells.^104^ The next step in establishing a functional *in vitro* conjunctival model is to develop a system derived from human conjunctival cells. Primary human conjunctival culture models are already used for conjunctival tissue transplantation.^105,106^ In addition, two immortalized human conjunctival cell lines have also become available and characterized,^107,108^ but have not yet been used to develop an *in vitro* model of human conjunctival epithelium.

## Retinal Morphology

**FIG. 4.**
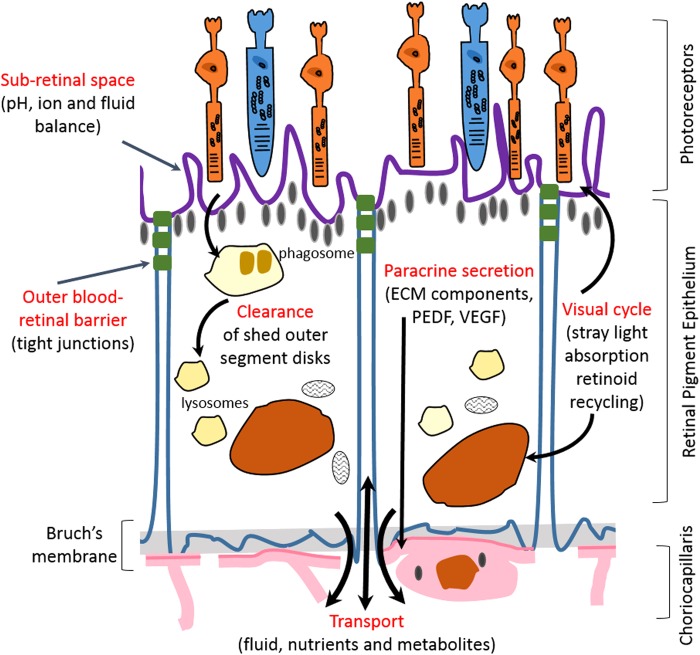
Schematic presentation of the functions of the retinal pigment epithelium within the retina.

The retina lines the inner surface of the eye, surrounds the vitreous cavity, and has an average thickness of 250 μm. It is designed to capture light and initiate processing of the image by the brain. The retina is protected and held in position by the surrounding sclera and cornea.^45^ The blood–retinal barrier (BRB) consists of three layers: the outer BRB, known as the RPE, the Bruch's membrane, and the inner BRB, which is the underlying choriocapillaris known as the retinal capillary endothelium^109^ (Fig. 4). The RPE is a tight monolayer of epithelial cells, which regulates the transport of nutrients and solutes, as well as the diffusion of systemic drugs from the choroid into the retina and vitreous humor.^110^ In contrast, the inner BRB is located in the inner retinal microvasculature and contains the microvascular endothelium that lines these vessels. Diffusion of many molecules from the blood to the retina is hindered by the tight junctions located between these cells, which are important in maintaining retinal homeostasis.^111^ Several cell culture models of the inner and outer BRB have been developed as useful tools for investigating cell biology and transport functions of RPE and retinal capillary endothelium (Table 4).

**Table 4.** **Cell Culture Models of Blood–Brain Barriers**

Species	Application(s)	References
Retinal pigment epithelium		
Primary isolated bovine cells	Assessment of barrier function	^116^
Primary isolated rat cells	Assessment of tight junctions	^114^
Immortalized rat RPE-J cell line	polarity and functions of the retinal pigment epithelium	^131^
Immortalized human cells (ARPE-19)	Toxicity, gene delivery, and polarity studies	^136,138,140^
Retinal capillary endothelium		
Primary isolated bovine retinal capillary (BRCEC)	Permeability studies	^147^
Immortalized rat retinal capillary endothelium	Barrier properties	^146,149^

## Retinal Models

### Retinal pigment epithelium

#### Primary models

Primary cell culture models of RPE derived from frog,^112,113^ rat,^114,115^ bovine,^116^ and chick^117^ have been widely described in the literature, however, most researchers are interested in models based on human RPE cells to avoid species-related applicability problems. Primary culture models of human RPE^118–120^ have been used for drug uptake and transport,^121–124^ protein expression,^125^ and cytotoxicity studies.^126–128^ As mentioned previously, there is limited availability of human donor eye cells in comparison with primary animal cells.

#### Immortalized models

To date, utilizing immortalized rat and human cell lines has led to different *in vitro* models of RPE being established.^67,129–132^ For instance, the rat RPE-J cell line with highly differentiated phenotype *in vitro* was developed by infection of rat primary RPE cells with a SV40 virus.^131^ In addition, the first spontaneously arising human RPE cell line was developed by Davis et al. from a primary culture of human RPE.^129^ The cells showed the ability to mimic metabolic and morphological properties of RPE cells *in vivo*, however, due to the loss of enzymatic activities and cytoskeletal polarization, the cell line is mainly used for cytotoxicity studies^133^ rather than for the assessment of drug permeation.^3^ Subsequently, Dunn et al.^130^ established and characterized the second human immortalized RPE cell line (ARPE-19) in terms of barrier properties and expression of retina-specific markers. ARPE-19 cells retained distinct cell borders and expressed retina-specific markers, RPE65 and CRLABP. In addition, due to the polarized distribution of cell surface markers, the authors suggested this model to be suitable for polarity studies in RPE cells.^130^ To more accurately reproduce the *in vivo* RPE phenotype, culture conditions of RPE cells have been altered, such as cocultivation with C6 glioma cells of immortalized astrocytes.^134^ However, RPE-19 cell lines did not grow in coculture or under conditioned medium mainly due to their heterogeneous nature.^4^ A third immortalized human RPE cell line was constructed by Bodnar et al., in which RPE cells were transfected by vectors encoding for human telomerase catalytic unit. As a result, the cellular life span was extended while cells retain normal growth characteristics and gene expression patterns.^132,135^

RPE-19 cell lines have been used by researchers for various *in vitro* experiments, such as toxicity studies,^136,137^ gene delivery,^138,139^ polarity studies of proteins,^140^ and as a model for retinal disease.^137,141^ However, the main limitation associated with models of RPE-19 cells is the long culture duration of up to 2 months for the cultured RPE cells to become more growth quiescent.^116,142^ In addition, due to the limited availability of human donor eyes, there are insufficient primary RPE cell culture models for comparison in the characterization of RPE immortalized cell lines and validating the *in vitro* culture models in terms of their *in vivo*/*in vitro* correlation. In addition, morphological and functional appearance of the RPE cultured cells is easily changed under cell culture conditions, a phenomenon known as deadaptation, and it is yet a challenge to develop a model of RPE that closely mimics many specialized features of the RPE.^143^

### Retinal capillary endothelium

Several pathogenic conditions of the eye such as diabetic retinopathy are related to the breakdown of the inner BRB.^144^ Therefore, understanding the underlying factors influencing the permeability of the retinal capillary endothelium is essential for discovering new treatment strategies for such diseases. To date, *in vitro* models of retinal capillary endothelium have been limited to primary isolated bovine retinal capillary endothelial cells (BRCEC)^145^ and immortalized rat retinal capillary endothelial cell line.^146^ Gillies et al. studied the permeability of insulin, expression of blood–brain barrier-related enzymes and effect of high glucose levels on the permeability of a BRCEC monolayer using bovine retinal capillary endothelial model.^147^ This model was further developed by Tretiach et al. as a coculture model of glial cells with primary BRCEC.^148^ However, this coculture model failed to grow as a monolayer, and also the barrier properties of the Gillies et al. model were not reproduced.^145^ In addition, Hosoya et al. developed a conditionally immortalized rat retinal capillary endothelial cell line,^146^ which was further evaluated by Shen et al. in terms of barrier properties.^149^ Such results concluded that so far, a retinal capillary cell culture model with *in vivo* barrier properties has not been developed. A better understanding of the *in vivo* barrier properties of the retinal capillary endothelium, detailed characterization of cell lines, and eventually coculture models will be necessary to establish and scientifically validate a more accurate *in vitro* model of the inner BRB.^3^

## Ocular Disease Models

To date, a number of animal and cell culture models of ocular diseases have been developed that help to investigate the molecular mechanism of ocular diseases and to screen ophthalmic drug candidates. Age-related macular degeneration (AMD) is a leading cause of blindness in people above the age of 60, which leads to visual impairment and a high rate of depression.^109^ Due to the unique features of the human eye and complexities associated with the nature of the disease, animal models fail to mimic all aspects of AMD.^150^ Therefore, cell culture models of RPE cells are useful alternative tools to investigate the physiology and pathology of the disease. In addition, cell culture models are advantageous because they are experimentally controlled systems and so the results are more reproducible than those obtained from animal models.^3^ A standard primary culture of AMD is human fetal RPE, which closely models the function and metabolic activity of native RPE.^151^ Other RPE cell models include RPE derived from stem cells and the immortalized ARPE-19 cell line.^130^

In addition, diseases of the optic nerve are also among the most devastating disorders in ophthalmology, which can result in the degeneration of retinal ganglion cell, visual field loss and, potentially, blindness. To date, the most useful glaucoma experimental animal models are monkeys, rats, and mice in which argon laser photocoagulation, diode laser photocoagulation, or translimbal laser photocoagulation are used to induce intraocular pressure. Although animal models are essential to improve our knowledge and to better understand the mechanism of each disease, developing an animal model for a disease is complex, challenging, and these animal models still differ widely in their applicability to the human disease.^152^ Therefore, there are a range of cell culture models of glaucoma, which include retinal ganglion cells, mixed retinal cells, transformed retinal cells, and neuronal-like cell lines. Once a culture model is established, multiple mechanisms can be used to simulate injury and study the effectiveness of neuroprotective therapies.^153^

## Vitreous Substitutes

The vitreous body is a gelatinous structure mainly composed of hyaluronic acid and different types of collagen (type II, IX, V/IX, and IV), which fills the space between the lens and the retina. The stability of the vitreous structure is mostly dependant on the presence of bound water to the glycosaminoglycans.^154^

Since 1960, clinical and bioengineering researchers have attempted to find an ideal vitreous humor substitute that would replicate two aspects of the native *in vivo* vitreous: on one hand, a substance with the same molecular structure to fill the ocular cavity and to mimic the elasticity and pressure within the eye, and on the other hand, presenting similar chemical and physiological characteristics of this gelatinous substance to allow perfusion of drugs and diffusion of metabolites.^155,156^ Some of the substances have been known for more than 20 years, however, others have been developed only recently to improve tolerability, stability, and tamponade effect (Table 5).

**Table 5.** **Vitreous Experimental Substitutes**

Types	Examples	Properties	References
Natural polymers	Hyaluronic acid and collagen	Great biocompatibility; short degradation time; low viscosity	^157,158^
Hydrogels	Poly(vinyl alcohol) Poly(1-vinyl-2-pyrrolidone), polyacrylamide	Great biocompatibility, stable transparency, and viscoelastic properties	^159–161^
Transplants and Implants	Artificial capsular bodies (foldable capsular vitreous body)	Good mechanical, optical, and biocompatible properties; may cause retinal disorders due to long-term capsule-induced mechanical pressure	^162,163^

## 3D *In Vitro* Models of the Eye

*In vitro* ocular cell culture models have been widely used in various fields of research; toxicological screening, permeability, studies of drug uptake and transport, cell physiology, and tissue engineering. They provide useful data that compliment findings from *in vivo* studies and allow significant reduction in the number of animals used. However, there are intrinsic restrictions associated with these models, mainly attributed to the fact that such systems are cell monolayers grown on a two-dimensional (2D) culture scaffold, which do not take into account the response of cells in the 3D curved environment present in the native ocular tissue.^164^ The *in vivo* 3D microenvironment can send signals to a cell through cell–cell or cell–extracellular matrix adhesion and mechanical forces.^40^ Consequently, these signals will activate a cascade of interactive events, which will in turn influence cell proliferation, differentiation, cellular structure morphology, and apoptosis.^40^ For instance, in drug discovery research, a 2D *in vitro* cell model that does not present accurate cellular properties and barrier functions may lead to selection of a candidate that cannot reach its target side of action *in vivo*. In addition, 3D cell culture provides more accurate depiction of cell polarization, since in 2D the cells can only be partially polarized. Moreover, 3D cell cultures have higher stability and longer lifespans than cell cultures in 2D. Also, 3D aggregates can be cultured for a longer period of time, at least up to 4 weeks, in comparison with almost 1 week in a 2D monolayer culture due to cell confluency.^40^ Therefore, 3D models are more appropriate for demonstrating long-term effects of the drug^36^ and to create robust and effective cell-based platforms in pharmaceutical research so that cellular responses will be more representative of those under *in vivo* conditions.^165^

None of the commercially available *in vitro* ocular models described in this review have been cultured on curved scaffolds to mimic growth conditions of corneal and retinal cells *in vivo*. In addition, ocular *in vitro* models are limited to regional parts of the eye and no model has yet been developed as an *in vitro* ocular equivalent as an organ. Only recently, a study by Postnikoff et al.^50^ has taken into account curved cell growth conditions, which focused on the creation of a 3D, stratified, curved epithelium. In this study, human papillomavirus-immortalized HCE cells were cultured on a curved Millicell-HA membrane (mixed cellulose esters; Millipore, Billerica, MA). This culture condition led to a stratified, curved, epithelial model suitable for assessment of cytotoxicity and biocompatibility testing of contact lenses.^50^ Therefore, the availability of accurate and informative 3D ocular *in vitro* models is an important challenge for applications in ophthalmic research, toxicity testing, and safety screening.

## Conclusion

Every year, around 50–100 million animals, ranging from zebra fish to nonhuman primates, are used worldwide in animal experiments. In 2010, the total number of animals used in the United States was almost 1.37 million, however, these statistics do not include rats and mice as these animals are not covered by the Animal Welfare Act in the United States, but still make up about 90% of research animals. In 2004, over 20,000 rabbits were used in the United Kingdom for the Draize eye irritancy tests and by 2011, over three million animals were generally used for experimentation, which mainly included mice (71%), fish (15%), rats (7%), and birds (4%).^166,167^ In view of this and to minimize the use of animals, a great amount of research has been dedicated to the development of nonanimal alternatives wherever necessary. Alternatives to animal studies are considered as anything from complete to partial replacement of live animals in biomedical research and experimental studies.^168^ An apparent 40% decrease in animal use and a simultaneous increase in the use of tissue culture and biotechnology show that scientifically valid nonanimal techniques are implementable.^169^

The development of alternative *ex vivo* ocular models has made important contributions to biological research. The BCOP and the ICE test methods have been in development since the early 1990s and are the first *ex vivo* ocular safety test methods that have been validated by the regulators. In both cases, the animal eyes used in both test methods are slaughterhouse waste, therefore animals were not specifically euthanized to obtain these tissues. William Stokes, director of NICEATM and executive director of ICCVAM, estimated that using these two assays alone could reduce the use of live animals for eye safety testing by 10% or more.^170^

In addition, the use of *in vitro* platforms has been greatly attributed to obvious cost and ethical advantages over *in vivo* models. Finally, the development of 3D *in vitro* culture models that more closely replicates *in vivo* and complements 2D cell culture and animal model findings, will help researchers to feel confident that final decisions based on *in vivo* ocular models are well supported.

## Abbreviations Used

2D: two-dimensional
3D: three-dimensional
AMD: age-related macular degeneration
BCOP: bovine cornea opacity and permeability
BRB: blood–retinal barrier
BRCEC: bovine retinal capillary endothelial cells
CE: corneal epithelium
EURL: European Union Reference Laboratory
EVCAM: European Center for the Validation of Alternative Methods
HCE: human corneal epithelial
IRE: isolated rabbit eye
LVET: low-volume eye irritation test
RPE: retinal pigment epithelium
SIRC: Statens Seruminstitut rabbit corneal cells
TEER: transepithelial electrical resistance
